# Primary Arthrodesis or Open Reduction and Internal Fixation for Lisfranc Injuries: A Systematic Review and Meta-analysis of Randomized Controlled Trials

**DOI:** 10.1177/24730114241286892

**Published:** 2024-10-18

**Authors:** Lachlan Mactier, Genevieve Cox, Rajat Mittal, Mayuran Suthersan

**Affiliations:** 1 University of Notre Dame Australia Rural Clinical School of Medicine, Darlinghurst, NSW, Australia; 2 Orthopaedic Department, Liverpool Hospital, Sydney, NSW, Australia; 3 Orthopaedics Department, Westmead Hospital, Sydney, NSW, Australia

**Keywords:** midfoot, Lisfranc, ORIF, arthrodesis

## Abstract

**Background:**

Lisfranc injuries are often managed surgically with primary arthrodesis (PA) or open reduction and internal fixation (ORIF); however, neither approach has been shown to be superior. This systematic review and meta-analysis assessed randomized controlled trials (RCTs) to compare the functional and surgical outcomes of PA and ORIF in the treatment of Lisfranc injuries.

**Methods:**

This study was performed as per the PRISMA protocol. Database searches were conducted on Cochrane, Embase, and PubMed libraries. Five RCTs were identified for inclusion involving 241 patients, of which 121 underwent PA and 120 underwent ORIF.

**Results:**

Statistically significant differences in visual analog scale pain score at 2 years (mean difference 0.89, 95% CI 0.18-1.59), patient satisfaction (OR 10.04, 95% CI 1.78-56.76), and all-cause return to surgery (OR 27.31, 95% CI 12.72-58.63) were observed, all favoring PA. There were no statistically significant differences between PA and ORIF with regard to American Orthopaedic Foot & Ankle Society midfoot scores at 2 years, 36-Item Short Form Health Survey (SF-36) scores, and unplanned return to surgery.

**Conclusion:**

This study showed significant improvement in pain at 2 years, patient satisfaction, and all-cause return to surgery favoring PA in all instances. Given ORIF often necessitates a second operation for hardware removal, it is to be expected that all-cause return to surgery is higher in ORIF groups. Overall, these results do not have the power to confer an advantage to a particular approach because of significant heterogeneity. Further studies should focus on larger patient cohorts and longer follow-up, or analysis stratified by patient demographics and injury presentation. In the absence of clinically significant differences, cost-benefit analyses should be considered to answer the question of whether to “fix or fuse” for Lisfranc injuries.

## Introduction

Lisfranc injuries are fracture dislocations of 1 or more of the metatarsals with respect to the medial cuneiform due to disruption of the interosseous Lisfranc ligament.^9^ Although being relatively rare, accounting for 0.2% of all fractures, it is estimated that more than 20% of these injuries are not identified on clinical examination or radiographs.^4,13^ Improperly treated Lisfranc injuries can lead to instability of the midfoot, chronic pain, or post-traumatic arthritis.^15,17,21^ Surgical management of Lisfranc injuries consists of either primary arthrodesis (PA) or open reduction and internal fixation (ORIF); however, neither approach has yet been shown to be superior.^1,8,12,22^

PA has previously been viewed as a salvage procedure in cases of polytrauma or post-traumatic arthritis.^10^ Conversely, ORIF is considered more appropriate for low-energy injuries and in younger populations. In the absence of long-term follow-up on the outcomes of PA, there is a tendency for ORIF to be the preferred approach where possible. The main drawback of this is the requirement for a second operation to remove hardware or conversion from ORIF to PA in cases of failed of healing.^2^

The purpose of this study is to review outcomes of PA and ORIF in the surgical management of Lisfranc injuries with a systematic review and meta-analysis. Previous systematic reviews and meta-analyses have included retrospective cohort (RC) studies and randomized controlled trials (RCTs)^1,8,12,22^; however, no literature to date has analyzed RCTs alone. This article seeks to build on previous research by including recently published works and only analyzing RCTs.

## Methods

This systematic review and meta-analysis was conducted per the Preferred Reporting Items for Systematic Review and Meta-Analysis (PRISMA).^11^

### Search Strategy

A search strategy was developed between all authors and implemented by 2 authors (L.M. and G.C.) independently. The databases formally searched were Cochrane, Embase, and PubMed; this was performed on July 3, 2024 (Appendix A). Additionally, a search was conducted in ClinicalTrials.gov (https://clinicaltrials.gov/ct2/home) and CENTRAL (https://www.cochranelibrary.com/central) databases using the term *lisfranc*.

### Literature Screening

**Figure 1.**
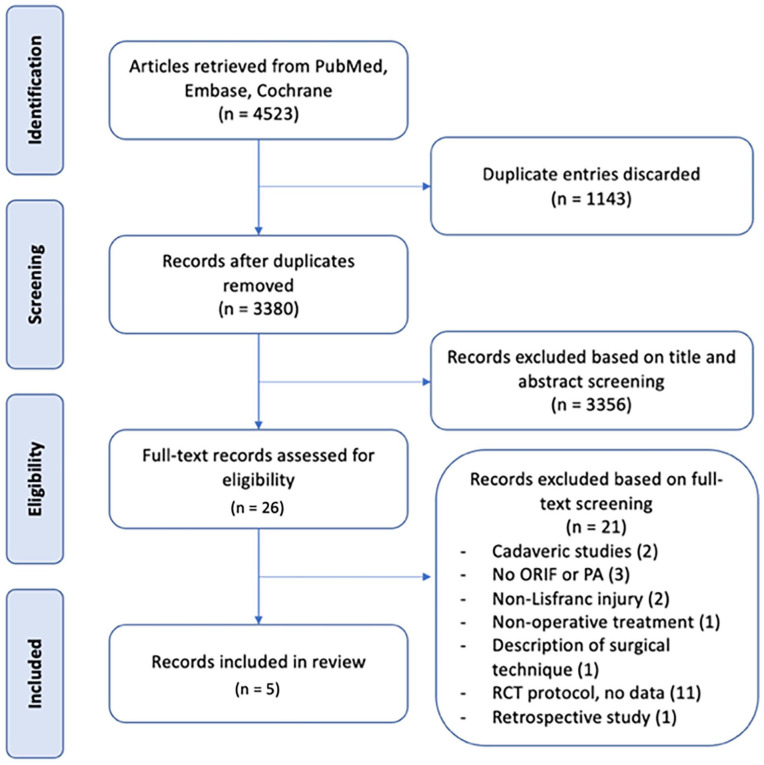
Flowchart of literature screening.

The results of the literature search were screened independently between 2 authors (L.M. and G.C.). All results of the search were initially screened by title and abstract for relevance to the research questions. Full-text screening of studies deemed relevant to the research question was performed and eligible articles were included for final analysis (Figure 1). Any discrepancies between results were discussed and resolved between the 2 authors conducting the search.

### Criteria for Study Inclusion

Subject to the search strategy, the following exclusion criteria were used in study selection: studies in nonhuman subjects, articles published in non-English language, studies other than randomized controlled trials only, and studies investigating patients who received an intervention occurred more than 8 weeks from the index Lisfranc injury.

### Risk of Bias Assessment

A risk of bias assessment was performed by one author (L.M.) on the studies included for analysis using the Cochrane Risk of Bias Assessment Tool (RoB2).^16^ This tool assesses each study on the parameters of the randomization process used, deviations in the study from the intended intervention, missing data outcomes, measurement of collected data, and selection of reported results. This identifies any articles that may have biases that could influence the outcome of results in a meta-analysis.

### Data Collection

Data were extracted from the included articles by one author (L.M.) and stored in Review Manager (RevMan) version 5.4 (The Cochrane Collaboration, 2020). Articles were labeled by lead author surname and year of publication within the application. Data were augmented using methodology described by Wan et al^20^ in instances where data from included studies were presented in nonstandard forms for meta-analysis and where appropriate information was available to impute data.

### Data Analysis

Data analysis was performed in RevMan using the Cochrane Handbook for Systematic Reviews of Interventions.^6^ For continuous variables, random effect modeling was performed, and the mean difference between PA and ORIF groups was calculated with a 95% CI. A positive mean difference favored PA for all variables, noting that the order of columns for visual analog scale (VAS) pain scores were reversed such that a positive mean difference indicated a lower VAS pain score. For dichotomous variables, fixed effect modeling was performed with an odds ratio (OR) comparing PA to ORIF calculated. An OR > 1 favored PA.

Heterogeneity was assessed for all variables and was determined if *P* ≤ .1 or interpreting *I*^2^, with *I*^2^ between 30% and 50% indicating moderate heterogeneity, 50 to 75% indicating considerable heterogeneity, and 75% to 100% indicating substantial heterogeneity.^3^

## Results

### Literature Search

A total of 3380 records were identified through the search strategy (Appendix A) after removal of duplicates. Of these, 26 studies were assessed as being relevant to the research question after title and abstract screening of all records, with further full-text review yielding 5 papers for inclusion in the meta-analysis (Figure 1). These studies involved 241 patients, 121 of whom underwent PA and 120 underwent ORIF. The characteristics of the included studies are detailed in Table 1. The results of the trials registry search are presented in Appendix B. Authors of these trials were contacted regarding the possibility of data to be included in this meta-analysis; however, no data were able to be collected.

**Table 1.** Characteristics of Included Studies.

Lead Author	Year of publication	Journal	Sample size	PA	ORIF	Mean Age, y	Gender (M/F)	Mean follow-up (mo)
Ly^7^	2006	*Journal of Bone & Joint Surgery*	41	20	21	NR	27/14	42.5
Henning^5^	2009	*Foot & Ankle International*	32	18	14	38.7	21/11	53
Stødle^18^	2020	*Foot & Ankle International*	48	24	24	32	22/26	NR
Sun^19^	2022	*International Orthopaedics*	78	38	40	40.7	50/28	37.8
Ponkilainen^14^	2024	*Foot & Ankle International*	43	21	22	34.4	27/16	NR

Abbreviations: M/F, male/female; NR, not reported; ORIF, open reduction and internal fixation; PA, primary arthrodesis.

### Risk of Bias Assessment

Of the 5 studies included in the analysis, 4 were assessed to have a low risk of bias (Table 2). One study (Ly and Coetzee^7^) was of some concern for bias because of the unmasked, quasi-random nature of the allocation method. In all 5 studies, the patients and the people performing the outcome assessments were not masked to which group the patient had been allocated postsurgery. This was deemed to be a low risk for bias as the outcomes measured were primarily standardized questionnaires (ie, American Orthopaedic Foot & Ankle Society [AOFAS] midfoot scale and 36-Item Short Form Health Survey [SF-36]) and objective dichotomous outcomes (eg, subsequent surgeries). All 5 studies had a high rate of follow through with the patients who were initially allocated to the studies.

**Table 2.** Risk of Bias Assessment for Included Studies.^a^

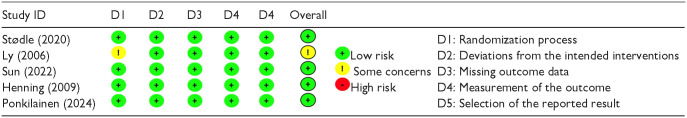

a Performed using the Cochrane tool to assess risk of bias in randomized controlled trials (RoB2). Green cells indicate a low risk of bias, yellow cells indicate some concern for bias and red cells indicate high risk of bias.

### AOFAS Midfoot Scale

**Figure 2.**
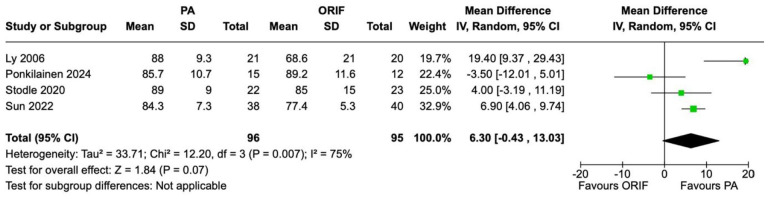
Forest plot for AOFAS midfoot scale scores at the 2-year follow-up comparing primary arthrodesis (PA) vs open reduction and internal fixation (ORIF).

AOFAS midfoot scale scores were recorded in 4 studies involving 191 patients (96 underwent PA and 95 underwent ORIF). There was no statistically significant difference observed between the PA and ORIF groups in mean AOFAS midfoot scale score 2 years postoperatively. The mean difference in AOFAS midfoot scale score at 2 years between the PA and ORIF groups was 6.30 (95% CI −0.43 to 13.03, *P* = .07). Substantial heterogeneity was observed between the included studies (*I*^2^ = 75%, *P* = .007) (Figure 2).

### SF-36

**Figure 3.**
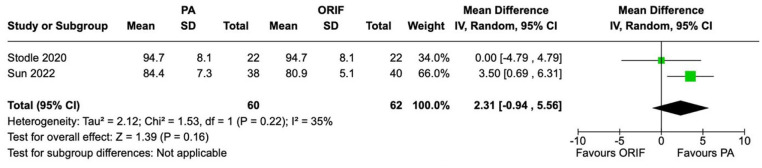
Forest plot for SF-36 physical function scores at the 2-year follow-up comparing primary arthrodesis (PA) vs open reduction and internal fixation (ORIF).

**Figure 4.**
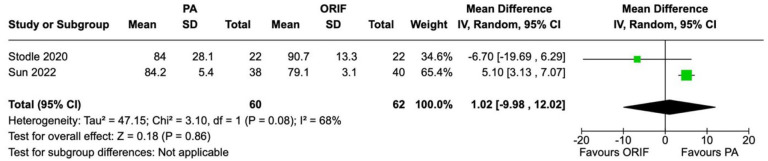
Forest plot for SF-36 bodily pain scores at the 2-year follow-up comparing primary arthrodesis (PA) vs open reduction and internal fixation (ORIF).

SF-36 scores were recorded in 2 studies involving 122 patients (60 underwent PA and 62 underwent ORIF). There was no statistically significant difference observed between the PA and ORIF groups in SF-36 physical function and SF-36 bodily pain. The mean difference in SF-36 physical function score between the PA and ORIF groups was 2.31 (95% CI −0.94 to 5.56, *P* = .16) and for SF-36 bodily pain was 1.02 (95% CI −9.98 to 12.02, *P* = .86). Moderate heterogeneity was observed between studies for SF-36 physical function (*I*^2^ = 35%, *P* = .22) and considerable heterogeneity for SF-36 bodily pain (*I*^2^ = 68%, *P* = .08) (Figures 3 and 4).

### VAS Pain

**Figure 5.**
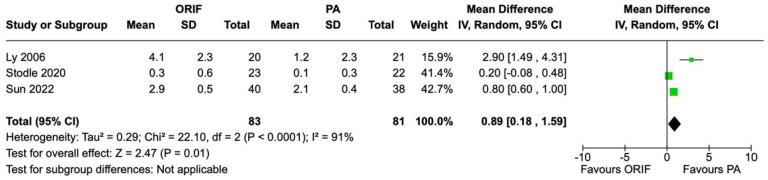
Forest plot for visual analog scale (VAS) pain scores at the 2-year follow-up comparing primary arthrodesis (PA) vs open reduction and internal fixation (ORIF).

VAS pain scores were recorded in 3 studies involving 164 patients (81 underwent PA and 83 underwent ORIF). Patients in the PA group had a statistically significant mean VAS score 0.89 points (95% CI 0.18-1.59, *P* = .01) lower than that of the ORIF group. Substantial heterogeneity was observed between studies for VAS pain (*I*^2^ = 91%, *P* < .0001) (Figure 5).

### Patient Satisfaction

**Figure 6.**
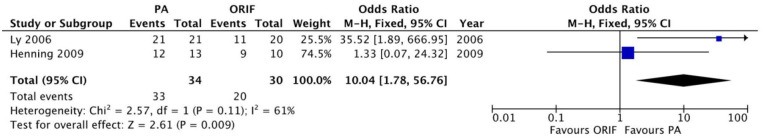
Forest plot for patient satisfaction (satisfied and very satisfied) comparing primary arthrodesis (PA) vs open reduction and internal fixation (ORIF).

Patient satisfaction was recorded in 2 studies involving 64 patients (34 underwent PA and 30 underwent ORIF). There was a statistically significant difference in the proportion of patients who reported being very satisfied or satisfied favoring the PA group over the ORIF group (OR = 10.04, 95% CI 1.78-56.76, *P* = .009). There was considerable heterogeneity observed between studies reporting patient satisfaction (*I*^2^ = 61%, *P* = .11) (Figure 6).

### Subsequent Surgery

**Figure 7.**
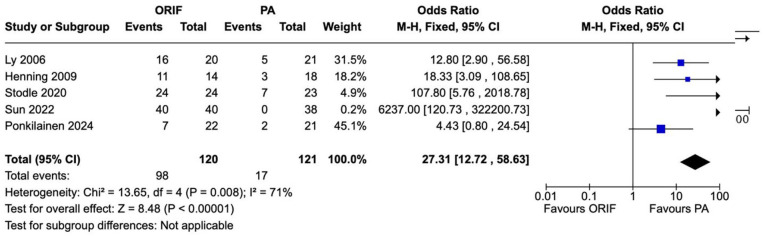
Forest plot for return to surgery (all cause) comparing primary arthrodesis (PA) vs open reduction and internal fixation (ORIF).

**Figure 8.**
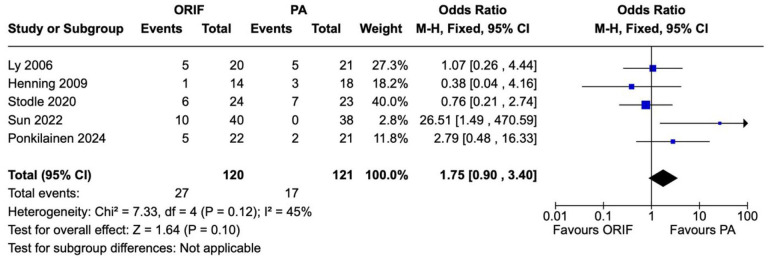
Forest plot for return to surgery (unplanned) comparing primary arthrodesis (PA) vs open reduction and internal fixation (ORIF).

Subsequent surgeries for any cause were reported in 5 studies involving 241 patients (121 underwent PA and 120 underwent ORIF). There was a statistically significant difference in subsequent surgeries of any cause between the PA and ORIF groups. The odds ratio between PA and ORIF groups was 27.31 (95% CI 12.72-58.63, *P* < .00001). Considerable heterogeneity was observed between the studies (*I*^2^ = 71%, *P* = .008) (Figure 7). There was no statistically significant difference in unplanned return to theater between the PA and ORIF groups (OR = 1.75, 95% CI 0.90-3.40, *P* = .10). There was moderate heterogeneity observed between the studies reporting unplanned return to theater (*I*^2^ = 45%, *P* = .12) (Figure 8).

## Discussion

We performed a systematic review and meta-analysis of randomized controlled trials investigating the use of primary arthrodesis or open reduction and internal fixation for Lisfranc injuries. The systematic review yielded 5 eligible RCTs for analysis including 241 patients, of which 121 underwent PA and 120 had ORIF. The outcomes measured were AOFAS midfoot scale scores, SF-36 scores, patient satisfaction scores, VAS pain scores, and return to surgery (planned and unplanned). This is the first systematic review and meta-analysis, to our knowledge, that only analyses RCTs that were intended to provide a definitive answer to the question of whether to fix or fuse primary Lisfranc injuries.

Our analysis showed statistically significant mean differences in VAS pain, patient satisfaction, and decreased likelihood for all-cause return to surgery, all favoring PA. Although the difference in VAS pain scores is significant, we suspect a difference of less than 1 point does not reach the minimal clinically important difference to warrant a change in practice in light of the heterogeneity observed between studies. Similarly, we suggest that the heterogeneity between the 2 studies, with small sample sizes, reporting patient satisfaction should not be used as a reason to choose a particular surgical approach. Furthermore, the significant difference in all-cause return to surgery between the PA and ORIF groups is to be expected given the common practice of follow-up surgery to remove hardware after ORIF of Lisfranc injuries. Without a significant difference in unplanned return to surgery between the PA and ORIF groups, it is difficult to assert that PA should be favored as the primary surgical management on the basis of fewer surgeries being required.

These results are concordant with previous systematic reviews and meta-analyses that have not produced compelling evidence to suggest either PA or ORIF is the superior surgical approach for management of Lisfranc injuries. We theorize that the reason neither approach has been found to be superior in any study to date is (1) there is significant heterogeneity in Lisfranc injuries in terms of severity and concomitant injuries; (2) the sample sizes presented in studies, RCT or otherwise, are limited; (3) the follow-up period required to demonstrate clinically significant outcomes exceeds that of published studies; or (4) the optimal approach may be dependent on factors such as severity of injury and range of ages and physical characteristics of patients rather than a one-size-fits-all approach.

With respect to the heterogeneity, we believe this arises from differences between the included studies with respect to treatment of tarsometatarsal (TMT) joints aside from the Lisfranc complex. To illustrate, in Stødle et al^18^ patients were randomized to PA or ORIF of the first TMT joint whereas the second and third TMT joints routinely were fused in both groups. Conversely, in Ly and Coetzee,^7^ the PA group underwent first and second TMT fusion (and third TMT in some cases), whereas the ORIF group routinely had ORIF of the first, second, and third TMT joints. The only commonality between all included studies was the treatment of the fourth and fifth TMT joints. We think this highlights the fact that it is difficult to identify the impact of the Lisfranc joint in the midfoot when there is dissimilar treatment of injured adjacent joints.

There are 3 main limitations to the study with respect to the data available for analysis that further limits the usefulness of these results. First, the included studies did not report the same outcome measures uniformly; thus for each outcome measure (except for subsequent surgeries) we were only able to analyze a subset of the 5 studies eligible for inclusion. This further narrowed the number of data points available for meta-analysis, limiting its overall power. Second, the data presented were not stratified according to factors such as patient demographics, injury mechanism, or injury pattern (ie, Myerson-Hardcastle grading), which prevented further subgroup analysis. Third, no distinction was able to be made between types of ORIF (eg, transarticular screw, Kirschner-wire fixation, or bridge plating) because of small sample sizes available in the included studies. We believe these are areas in which future works could improve in order for treatment algorithms to be developed to direct specific surgical intervention.

In light of this, it is still unclear as to what the most appropriate surgical management of Lisfranc injuries is, although it must be considered that both approaches may in fact produce equivalently acceptable results. Without large population data with long-term follow-up, in the order of 10-15 years, we would suggest that additional RCTs similar to those published will not add evidence base on this topic. Studies into the cost-effectiveness of PA vs ORIF or whether a particular approach should be preferred given patient demographics have not yet been published. Given the lack of compelling evidence for PA or ORIF, studies investigating these parameters may present a better rationale for a particular approach to be preferred at this time.

## Conclusion

There is no consensus on whether to perform open reduction and internal fixation or primary arthrodesis to treat Lisfranc injuries. This systematic review and meta-analysis of RCTs on operative treatment of Lisfranc injuries showed statistically significant improvements in VAS pain score at 2 years and patient satisfaction and reduced all-cause return to surgery favoring primary arthrodesis over open reduction and internal fixation. However, given significant heterogeneity in the data presented, it cannot be asserted that primary arthrodesis is superior to internal reduction and open fixation in all cases. We advocate for future studies that focus on stratifying patients based on factors such as injury mechanism, injury pattern, and demographics Alternatively, studies that analyze treatment from a cost-benefit perspective may be more useful if clinical differences between approaches do not in fact exist. In the absence of compelling evidence to support either approach, it seems most appropriate the decision to “fix or fuse” be based on clinical judgement until more nuanced, data-driven treatment algorithms are developed.

## Supplemental Material

sj-pdf-1-fao-10.1177_24730114241286892 – Supplemental material for Primary Arthrodesis or Open Reduction and Internal Fixation for Lisfranc Injuries: A Systematic Review and Meta-analysis of Randomized Controlled Trials Supplemental material, sj-pdf-1-fao-10.1177_24730114241286892 for Primary Arthrodesis or Open Reduction and Internal Fixation for Lisfranc Injuries: A Systematic Review and Meta-analysis of Randomized Controlled Trials by Lachlan Mactier, Genevieve Cox, Rajat Mittal and Mayuran Suthersan in Foot & Ankle Orthopaedics

## Appendix A

### Databases

• PubMed, Embase, Cochrane

### Search Criteria

• Exclude result >50 years old from date of search
• Only results published in English
• Exclude nonhuman studies
• Exclude non-RCT studies

**Table:**

Concept 1: Affected Area	Concept 2: Injury Type	Concept 3: Surgical Intervention	Concept 4: Surgical Intervention
• Lisfranc • Metatarsal* • Tarsometatarsal • Cuneometatarsal • Intercuneiform • Midfoot • Forefoot	• Injury* • Fracture • Dislocation* • Trauma*	• Primary fixation • Fixation • Plate/plating • ORIF • Open reduction (and) internal fixation • Nonfusion • Fracture fixation* • Fracture fixation, internal*	• Fusion • Joint fusion • Primary arthrodesis • Arthrodesis*

* Indicates MeSH term in PubMed and Embase.

Concept 1: ((lisfranc) OR (metatarsal) OR (tarso-metatarsal) OR (cuneo-metatarsal) OR (inter-cuneiform) OR (midfoot) OR (forefoot))
Concept 2: ((injury) OR (fracture) OR (dislocation) OR (trauma))
Concept 3: ((primary fixation) OR (fixation) OR (plating) OR (ORIF) OR (open reduction) OR (internal fixation) OR ((open reduction) AND (internal fixation)) OR (non-fusion) OR (fracture fixation) OR (fracture fixation, internal))
Concept 4: ((fusion) OR (joint fusion) OR (arthrodesis) OR (primary arthrodesis))
Concept 5: ((rct) OR (randomized control trial) OR (randomized)

### PubMed Search Strategy (RCT option selected manually)

#1: Concept 1
#2: Concept 1 AND Concept 2
#3: Concept 1 AND Concept 3
#4: Concept 1 AND Concept 4
#5: Concept 1 AND Concept 3 AND Concept 4
#6: Concept 2 AND Concept 4
#7: Concept 2 AND Concept 3 AND Concept 4
#8: Concept 3 AND Concept 4

### Embase Search Strategy (RCT filtered)

#1: Concept 1
#2: Concept 1 AND Concept 2
#3: Concept 1 AND Concept 3
#4: Concept 1 AND Concept 4
#5: Concept 1 AND Concept 3 AND Concept 4
#6: Concept 2 AND Concept 4
#7: Concept 2 AND Concept 3 AND Concept 4
#8: Concept 3 AND Concept 4

### Cochrane Search Strategy (Selected Trials and English)

#1: Concept 1
#2: Concept 1 AND Concept 2
#3: Concept 1 AND Concept 3
#4: Concept 1 AND Concept 4
#5: Concept 1 AND Concept 3 AND Concept 4
#6: Concept 2 AND Concept 4
#7: Concept 2 AND Concept 3 AND Concept 4
#8: Concept 3 AND Concept 4

## Appendix B

**Table 1.** Unpublished studies identified as potentially having data for inclusion. BMC (BioMed Central), ANZCTR (Australian New Zealand Clinical Trials Registry), ChiCTR (Chinese Clinical Trials Registry). Authors were contacted but no data were able to be collected.

Lead Author	Year of Publication or Trial Registration	Journal or Registry	Country	Title
**Van den boom**	2021	BMC Surgery	Netherlands	The BFF Study: The Better to Fix or Fuse Study 'Retaining or Removing the Joint in the Foot: A Randomized Controlled Multicenter Trial of Primary Arthrodesis Versus Joint Stabilisation in Lisfranc Fracture Dislocation Midfoot Injuries.
**D’Arcy**	2022	ANZCTR	New Zealand	Management of the Absolutely Ligamentous Lisfranc injury: Early fusion vs fixation Treatment: the mini-MALLET trial; a pilot randomized controlled trial.
**Bilenki**	2019	ANZCTR	Australia	Midfix RCT: Does dorsal plating of Lisfranc injuries lead to better MOxFQ pain scores at 1 year compared to transarticular screw fixation? – A prospective randomized controlled trial.
**Zhang**	2018	ChiCTR	China	Primary arthrodesis compared with open reduction and internal fixation for Lisfranc injuries.
